# Can low-dose methotrexate reduce effusion-synovitis and symptoms in patients with mid- to late-stage knee osteoarthritis? Study protocol for a randomised, double-blind, and placebo-controlled trial

**DOI:** 10.1186/s13063-020-04687-3

**Published:** 2020-09-16

**Authors:** Zhaohua Zhu, Qinghong Yu, Xiaomei Leng, Weiyu Han, Zhanguo Li, Cibo Huang, Jieruo Gu, Yi Zhao, Kang Wang, Tianwang Li, Yifang Mei, Jianhua Xu, Zhiyi Zhang, David Hunter, Flavia Cicuttini, Xiaofeng Zeng, Changhai Ding

**Affiliations:** 1 Clinical Research Centre, Zhujiang Hospital, Southern Medical University, Guangzhou, Guangdong China; 2 Department of Rheumatology and Clinical Immunology, Zhujiang Hospital of Southern Medical University, Guangzhou, China; 3 Menzies Institute for Medical Research, University of Tasmania, Hobart, Tasmania Australia; 4 Department of Rheumatology, Peking Union Medical College Hospital, Chinese Academy of Medical Sciences & Peking Union Medical College, Beijing, China; 5 Department of Rheumatology & Immunology, Peking University People’s Hospital, Beijing, China; 6 Department of Rheumatology, Beijing Hospital, National Center of Gerontology, Beijing, China; 7 Department of Rheumatology, 3rd Affiliated Hospital of Sun Yet-Sen University Guangzhou, Guangzhou, Guangdong China; 8 Department of Rheumatology & Allergy, Xuanwu Hospital, Capital Medical University, Beijing, China; 9 Department of Rheumatology, the First Affiliated Hospital of Anhui Medical University, Hefei, Anhui China; 10 Department of Rheumatology and Immunology, Guangdong Second Provincial General Hospital, Guangzhou, China; 11 Department of Rheumatology, the First Affiliated Hospital of Harbin Medical University, Harbin, China; 12 Department of Rheumatology, Royal North Shore Hospital and Institute of Bone and Joint Research, Kolling Institute, University of Sydney, Sydney, Australia; 13 Department of Epidemiology and Preventive Medicine, Monash University, Melbourne, Australia

**Keywords:** Methotrexate, Osteoarthritis, Effusion-synovitis, Pain

## Abstract

**Background:**

Osteoarthritis (OA) is a common chronic disease in older adults. Currently, there are no effective therapies to reduce disease severity and progression of knee OA (KOA), particularly in mid- to late-stages. This study aims to examine the effect of methotrexate (MTX) on knee effusion-synovitis and pain in symptomatic patients with mid- to late-stage KOA.

**Methods/design:**

This protocol describes a multicentre randomised placebo-controlled clinical trial aiming to recruit 200 participants with mid- to late-stage symptomatic KOA and with effusion-synovitis grade of ≥ 2. Participants will be randomly allocated to the MTX group (start from 5 mg per week for the first 2 weeks and increase to 10 mg per week for the second 2 weeks and 15 mg per week for the remaining period if tolerated) or the placebo group. Primary outcomes are effusion-synovitis size measured by magnetic resonance imaging (MRI) and knee pain assessed by visual analogue scale (VAS). Secondary outcomes are signal intensity alteration within infrapatellar fat pad (IPFP) and Western Ontario and McMaster Universities Osteoarthritis Index (WOMAC) total score and subscores, and the Outcome Measures in Rheumatology Arthritis Clinical Trials-Osteoarthritis Research Society International (OMERACT-OARSI) responders. Both intention-to-treat and per-protocol analyses will be performed.

**Discussion:**

If MTX intervention can relieve symptoms and reduce inflammation in patients with mid- to late-stage KOA, it has the potential for significant clinical and public health impact as this low-cost and commonly used intervention would delay the time to knee replacement, leading to substantial cost savings and improve quality of life.

**Trial registration:**

ClinicalTrials.gov NCT03815448. Registered on 21 January 2019.

## Introduction

Osteoarthritis (OA) is one of the commonest chronic conditions in older people. It is characterised by gradual loss of articular cartilage and changes of other joint tissues, eventually leading, in some cases, to total joint replacement [1]. Approximately 25% of people over 55 years old have had knee pain on most days in a month in the past year. Of these, about half have radiographic knee OA (KOA) and are considered to have symptomatic KOA [1]. In China, the prevalence of symptomatic KOA was estimated up to 8.1% among people of 45 years and older [2]. There are more than 1 million hip or knee replacements every year in the USA, and the number is continuing to rise [3]. The costs of treating OA are expected to grow with our increasing ageing population.

Current treatments for knee OA are limited as there are no effective therapies to reduce disease severity and progression of knee OA particularly in mid- to late-stages [4]. People with mid- to late-stage OA often live with severe pain and have significant difficulty to perform daily activities [5], leading to substantial physical and mental health consequences and diminished quality of life [6]. There is, therefore, an urgent need to find better ways to manage symptoms and disease progression in mid- to late-stage OA.

There are a number of different phenotypes with different etiologies [7]. One is manifested by the presence of synovitis [8]. Pathological studies have shown that synovitis presence is associated with alterations in the adjacent cartilage that are similar, though to a lesser extent, to those seen in rheumatoid arthritis (RA) [9]. Joint effusion shown on non-contrast enhanced T2- or proton density-weighted MRI reflects a composite of true joint effusion (i.e. joint fluid) and synovial thickening [10], which has been regarded as effusion-synovitis. MRI-detected effusion-synovitis strongly predicted the development of incident radiographic OA over 4 years in the Osteoarthritis Initiative (OAI) study [11]. Suprapatellar pouch effusion-synovitis grade or maximal area was associated with increased knee pain, loss of knee cartilage volume, and increase in cartilage defects and bone marrow lesions (BMLs) [12, 13].

Low-dose methotrexate (MTX) administered weekly is commonly prescribed in patients with inflammatory arthritis including RA due to its anti-inflammatory effect and has good long-term safety [14]. There is evidence that low-dose MTX may have beneficial effects on OA via a variety of mechanisms. In a lapine model of OA, treatment with MTX for 12 weeks significantly reduced cartilage lesions in the femoral condyles [15]. In a single-centre, open-label study with 30 knee OA patients, MTX treatment (commenced from 7.5 mg, increasing up to 20 mg at 6 weeks) for 24 weeks significantly reduced knee pain assessed by visual analogue scale (VAS) and the Western Ontario and McMaster Universities Osteoarthritis Index (WOMAC); its analgesic efficacy was comparable to that achieved with NSAIDs and even opioids [16]. A recent multicentre randomised, double-blinded, placebo-controlled trial recruited 155 patients with symptomatic knee OA to examine if MTX treatment (*20–25 mg weekly*) for 6 months could reduce symptoms and disease progression. Preliminary results showed that compared with placebo, MTX modestly reduced knee pain assessed using Numeric Rating Scale, but had no effects on WOMAC knee pain [17]. It had no effects on MRI-assessed knee synovitis [17]. However, this clinical trial has some limitations: (1) the primary outcome evaluation was short (6 months) and may not be long enough to observe disease progression, (2) patients were not selected on the basis of the presence of inflammation, and (3) synovitis was not used as the primary and/or secondary outcomes.

Thus, taken together, there is evidence to suggest that MTX treatment is associated with reduced joint effusion and knee pain, indicating that MTX may have disease-modifying as well as symptom-relieving effects on knee OA. MTX may be particularly acceptable to those with mid- to late-stages of knee OA and joint effusion-synovitis, who have failed other OA therapies.

## Methods and design

### Trial aims

#### Primary aims

The primary aim is to examine, in a randomised, double-blind, and placebo-controlled trial over 52 weeks, whether low-dose MTX reduces knee joint effusion-synovitis and VAS pain compared to placebo in patients with mid- to late-stage knee OA and effusion-synovitis.

#### Secondary aims

The secondary aim is to examine, in a randomised, double-blind, and placebo-controlled trial over 52 weeks, whether low-dose MTX reduces IPFP signal intensity alteration (Hoffa’s synovitis); improves WOMAC pain, function, and stiffness; and has higher OMERACT-OARSI responders compared to placebo in patients with mid- to late-stage knee OA and effusion-synovitis.

#### The primary hypotheses

Compared to placebo, MTX treatment (up to 15 mg per week) over 52 weeks in patients with mid- to late-stage knee OA and effusion-synovitis will:

1. Reduce joint effusion-synovitis size assessed by MRI
2. Improve knee pain assessed by VAS

#### The secondary hypotheses

Compared to placebo, MTX treatment (up to 15 mg per week) over 52 weeks in patients with mid- to late-stage knee OA and effusion-synovitis will reduce IPFP signal intensity alteration (Hoffa’s synovitis) assessed by MRI; improve WOMAC pain, function, and stiffness; and have higher proportion of OMERACT-OARSI responders.

### Study design

This study is designed as a multicentre, randomised, double-blind, placebo-controlled trial over 52 weeks. The study will be conducted with adherence to the CONSORT guidelines. Two hundred participants from academic hospitals with mid- to late-stage symptomatic knee OA and effusion-synovitis will be recruited and randomly allocated to the treatment or placebo control group. The recruitment strategy will include collaborations with specialist rheumatologists from the following 9 sites: Zhujiang Hospital of Southern Medical University, Peking Union Medical College Hospital, Peking University People’s Hospital, Beijing Hospital, 3rd Affiliated Hospital of Sun Yet-Sen University Guangzhou, Xuanwu Hospital of Capital Medical University, the First Affiliated Hospital of Anhui Medical University, Guangdong Second Provincial General Hospital, and the First Affiliated Hospital of Harbin Medical University. Ethical approval has been received from Ethics Committee of Zhujiang Hospital of Southern Medical University (reference number: 2018-FSMYK-001).

### Participants

**Fig. 1:**
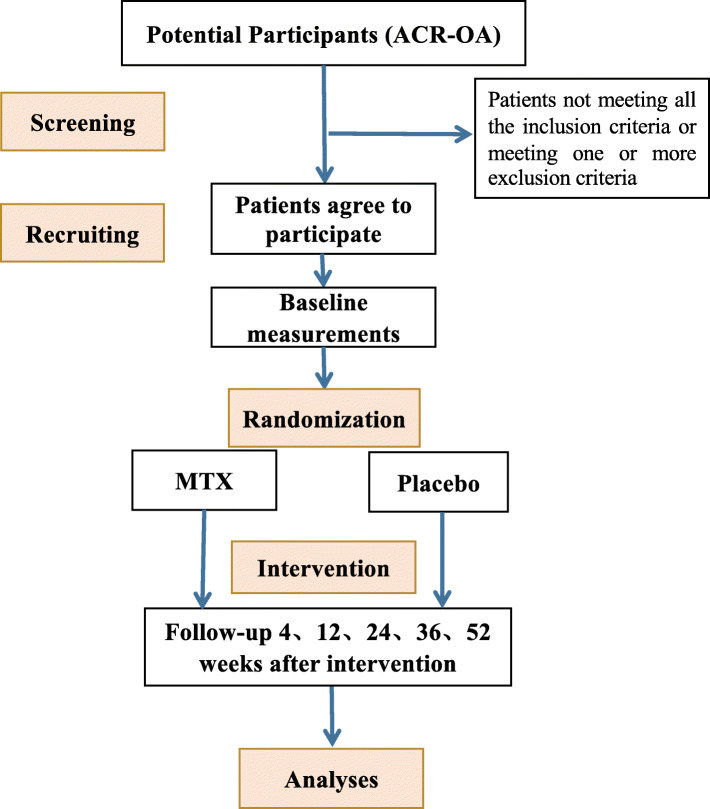
Flow chart of trial participation

At screening, participants will complete questionnaires, clinical examinations, blood tests (blood cell counts, liver function, renal function, hepatitis B/C tests), and chest X-ray and have a knee X-ray and MRI to ensure the inclusion/exclusion criteria are met. The index knee will be defined as the one with symptomatic OA; if both are symptomatic, the one with more severe knee symptoms will be used as the index knee. There will be 7 study visits (screening, baseline, and weeks 4, 12, 24, 36, and 52) (Fig. 1). The study measures are shown in Table 1. If the participant withdraws after a minimum of 24 weeks’ treatment, he/she will be requested to have a second knee MRI scan.

**Table 1:** Timetable and measures to be made

	Screening	Weeks						Withdraw
		0	4	12	24	36	52	
***Primary outcomes***								
MRI (effusion-synovitis volume)	✔						✔	✔
Knee pain (VAS)	✔		✔	✔	✔	✔	✔	✔
***Secondary outcomes***								
MRI (IPFP intensity alteration)	✔						✔	✔
WOMAC	✔		✔	✔	✔	✔	✔	✔
***Other measures***								
Medical history	✔							
Clinical assessment	✔							
MRI (effusion-synovitis score)	✔						✔	✔
X-ray (knee)	✔							
X-ray (lung)*	✔							
Blood tests	**✔**		✔	✔	✔	✔	✔	✔
General health status (VAS)	✔		✔	✔	✔	✔	✔	✔
SF-12	✔		✔	✔	✔	✔	✔	✔
PHQ-9	✔		✔	✔	✔	✔	✔	✔
Weight/height	✔							
Waist/hip circumference	✔							
Medication		✔	✔	✔	✔	✔	✔	✔
Tablet number		✔	✔	✔	✔	✔	✔	✔
Adverse event		✔	✔	✔	✔	✔	✔	✔

*Up to the principal investigators to decide if necessary

### Inclusion criteria

The inclusion criteria are as follows: (1) men and women, aged 45–70 years old; (2) meet the American College of Rheumatology (ACR) criteria for clinical knee OA assessed by a rheumatologist (knee pain + at least 3 of 5 that not including “no palpable warmth”) [18]; (3) knee pain on most days for at least 6 months, with a pain VAS of at least 40 mm; (4) Kellgren-Lawrence grade of 2 to 4; (5) physical examination for signs of inflammation (at least 2 of the following 4 clinical signs of inflammation: warmth over the joint area, joint margin tenderness, synovial effusion, and soft tissue swelling around the knee); (6) MRI-assessed effusion-synovitis grade of ≥ 2; and (7) able to provide written, informed consent.

### Exclusion criteria

The exclusion criteria were as follows: (a) any inflammatory arthritis (e.g. gout, reactive arthritis, rheumatoid arthritis, psoriasis, psoriatic arthritis, seronegative spondyloarthropathy) or systemic lupus erythematosus; (b) previous or planned knee replacement or surgery including arthroscopy within 12 months; (c) severe valgus knee deformity (angle of genu valgum > 30°); (d) contraindication to MRI; (e) use of intra-articular, intra-muscular, or oral corticosteroids in previous 4 weeks; (f) use of other anti-synovitis agents (e.g. hydroxychloroquine or sulphasalazine) in previous 3 months; (g) any clinically significant condition(s) such as (but not limited to) active cancer including lymphoma, and renal (e.g. abnormal renal function), hepatic (including active hepatitis B, hepatitis C, abnormal liver function), severe respiratory (e.g. lung infection, pulmonary fibrosis), haematological (e.g. white cell count < 4 × 10^9^/L, platelets < 100 × 10^9^/L, or haemoglobin < 100 g/L), gastrointestinal, endocrine, cardiac, neurologic, or cerebral diseases; (h) history of infections such as syphilis and HIV; (i) highly allergic to MTX; and (j) pregnant or lactating women.

### Randomisation/blinding

Allocation of participants will be based on computer-generated random numbers using block randomisation. To control for variation in centres, the randomisation will be stratified according to the centres. The RCT will be blinded to participants, investigators, and those measuring the effect of the intervention. Allocation concealment will be ensured by the use of a central automated allocation procedure, with security in place to ensure allocation data cannot be accessed or influenced by any person. Participants will be assigned to the interventions after baseline screening. Sequentially numbered and sealed opaque envelopes which reveal random numbers will be provided to the centres. Identical drugs for each participant will be allocated by research nurse base on the corresponding random number. Objective measures of knee structural changes and knee pain will be made by trained observers who are blinded to group allocation. The statistician who performs the statistical analyses will also be blinded. Unblinding will be allowed in emergency that affects the safety of participants. We will withdraw the unblinded participants but will continue to follow-up as per planned schedule.

### Intervention

After randomisation, participants in the intervention group will receive MTX (start from 5 mg per week for the first 2 weeks and increase to 10 mg per week for the second 2 weeks and 15 mg per week for the remaining period if tolerated). Plasma levels of oral MTX in a dose of 15 mg per week were similar as in a dose of 20 mg or 25 mg per week [19], so a maximal dose of 15 mg per week will be used in this study. The dose can be modified at the rheumatologist’s discretion regarding its intolerance or laboratory abnormalities [e.g. medication will be ceased if aspartate or alanine aminotransferase (AST, ALT) level greater than 3 times the upper limit of normal, or if AST or ALT level greater than twice the upper limit of normal on 2 continuous occasions; the dose will be reduced until 5 mg if AST or ALT level is raised but does not reach the level mentioned above]. The control group will receive an identical inert placebo per week. To reduce possible side effects, weekly doses of folic acid 5 mg will be given 1 day after MTX usage. This dose of folic acid is safe and may have additional benefits such as cardiovascular reduction [20]. Because skin dryness, skin rashes, and increased risk of skin malignancy (esp melanoma) may occur, the participants will be advised to wear a hat when out in the sun. Alcohol is avoided while on this medication. “Patient information on MTX” from the Chinese Rheumatology Association will be provided to all participants.

During the trial, all participants will not be asked to stop their analgesic medications, so specific medication will be recorded including the dosage and duration. However, corticosteroids may significantly affect the evaluation of outcomes, so their use will not be allowed. Trimethoprim (*Bactrim or Septra*), an antibiotic often used for respiratory and urinary infections, may increase the toxicity of MTX, so will also be disallowed in the trial. Participants who do not benefit from current study post-trial will be referred to appropriated outpatient treatments.

### Quality assurance

All research staff will be trained using the standard protocol ahead of the recruitment. The trial will be delivered in accordance with the protocol, and case report forms will be provided to the site staff. The investigators, research assistants, and outcome assessors are different people. Protocols will not be altered during the study timeframe.

### Outcome measures

The primary endpoints of the study will be effusion-synovitis volume and VAS pain. The secondary endpoints of the study will be IPFP signal intensity alteration measures, WOMAC total score and subscales, and OMERACT-OARSI responders.

#### Assessment of MRI structural changes

Knees will be imaged in the sagittal plane on a 3.0-T whole-body magnetic resonance unit with use of a commercial transmit-receive extremity coil. T2-weighted fast spin-echo and fat-saturated T1-weighted spoiled gradient echo sequences will be used. Changes in effusion-synovitis and IPFP signal intensity measures from baseline to week 52 are the endpoints, but we will also assess changes in total scores of cartilage defects and BMLs.

1. *Knee joint effusion-synovitis:* Knee effusion-synovitis maximal volume/area [13] at suprapatellar pouch will be measured at screening and week 52, and severity will be scored from 0 to 3 according to the estimated maximal distension of the synovial cavity by assessing the amount of intra-articular fluid-equivalent signal on T2-weighted MRI [21]. Effusion-synovitis at central portion, posterior femoral recess, and subpopliteal recess will also be assessed.
2. *IPFP signal intensity alterations:* Signal intensity alterations within IPFP were defined as discrete areas of increased signal. Standard deviation of IPFP signal intensity[sDev(IPFP)] and Clustering Factor(H) will be used to represent the signal intensity alteration within IPFP. The measurements will be performed using sagittal planes of fat-saturated T2-weighted images [22] at screening and week 52.

#### Assessment of knee symptoms

Knee pain will be assessed by VAS (0–100) and Western Ontario and McMaster Universities Osteoarthritis Index (WOMAC) [23] at each time point. Knee function and stiffness will also be assessed using WOMAC. The WOMAC consists of 24 items covering three subscales: pain (5 items), stiffness (2 items), and physical function (17 items). Each subscale will be transformed to a score ranging from 0 to 10, with a higher score indicating greater pain and physical function impairment.

#### Other measures

1. *Cartilage defects:* The cartilage defects will be graded at screening and week 52 on T2-weighted MR images using a modified Outerbridge classification at medial tibial and femoral, lateral tibial and femoral, and patellar sites from grade 0 to 4. Intra-observer reliability (expressed as ICC) was 0.89–0.94, and inter-observer reliability from previous studies was 0.85–0.93 [18].
2. *Subchondral BMLs:* The subchondral BMLs will be assessed at screening and week 52 on T2-weighted MR images and defined as discrete areas of increased signal adjacent to the subcortical bone at the lateral, medial femur and/or tibia. Based on the modified Whole-Organ Magnetic Resonance Imaging Score (WORMS) method [24], each BML will be scored on the basis of lesion size from grade 0 to 3 (if present on > 3 consecutive slices). The intraclass correlation coefficients (ICCs) in different sites from a previous study were 0.89–1.00 [25]. We have also developed BML maximum area as a sensitive measure of progression [26].
3. *Radiographic OA:* This will be assessed at screening by a standing semiflexed anteroposterior radiograph of the diseased knee. Radiographic OA status will be assessed using Kellgren-Lawrence (grade 0–4). X-rays will also be scored for osteophytes and joint space narrowing on a 4-point scale (0–3) by two simultaneous observers utilising the OARSI atlas. This method has excellent reproducibilities with an ICC of 0.99 for osteophytes and 0.98 for joint space narrowing [27].
4. *Angle of genu valgum:* This will be measured from weight-bearing radiographs at screening. Our intra-observer correlation coefficient was 0.98 [28].
5. *General characteristics:* Age and sex will be recorded at each time point except screening. Height (stadiometer), weight (electric scales), and waist and hip circumference will be measured using standard procedures.
6. *Quality of life:* The Short Form 12 (SF-12) item questionnaire which includes measures of physical function, general health, mental health, role-physical and role-motional, social functioning, vitality, and bodily pain level will be used to assess quality of life [29] at each time point except screening.
7. *Depression*: The Patient Health Questionnaire-9 (PHQ-9) will be used at each time point except screening to measure and monitor the depression of participants [30].
8. *Cigarette smoking status, previous knee injury, and occupation* will be assessed by questionnaires at baseline and week 52 [31].
9. *Concomitant medication usages* such as glucosamine, chondroitin, and non-steroid anti-inflammatory drugs will be recorded by questionnaire at each study visit.

### Safety assessments

The safety profile of MTX has been studied for over 25 years, it is well tolerated by most patients in the weekly low doses used for RA treatment, and it is a well-known drug with a well-established side effect profile. Most patients do not experience side effects, and for those who do, many of the minor side effects will improve with time (American College of Rheumatology Guideline for MTX). Adverse events of MTX will be closely monitored at each visit during the study. The most common side effects include nausea, malaise, vomiting, diarrhoea, and stomach pain; mouth ulcers; skin dryness, skin rashes, and increased sensitivity to the sun; mild tiredness, headache, and mental clouding; and temporary increase in muscle and joint pain. Less common or rare possible side effects include infection, cytopenia, abnormal liver function with fibrosis, and non-infectious pneumonitis (presenting with dry cough, shortness of breath, ± fever). Should any serious adverse events (SAEs) occur, they will be recorded on a separate form and principal investigators (PIs) will be notified within 24 h. An independent safety monitoring board will be convened, consisting of two clinical rheumatologists, a clinical pharmacologist experienced in MTX use, and a biostatistician, all with clinical trial experiences. They will meet bimonthly or more often if SAEs occur, and provide a written report to the PIs. The dose of MTX will be modified based on the tolerance at the rheumatologist’s discretion.

### Sample size

The sample size calculations were performed based on formulae provided by Cohen [32] and based on the following parameters: (1) *α* = 0.05, 2-sided; (2) *β* = 0.20, power = 80%; and (3) our previous study reported that suprapatellar pouch effusion maximum area in older people with knee pain was 2.0 ± 1.5 cm^2^. To determine an expected rate of effusion-synovitis decrease after MTX treatment of 35% [33], the calculated sample size will be 74 per arm (Table 2). A study from our group reported that patients with knee OA had a VAS knee pain score (0–100) of 50 ± 20 [26]. Assuming knee pain in OA patients will be reduced by 20% following MTX treatment, the sample size needed to detect these differences is 64 per group (Table 2). A decrease in knee pain of 20% or more is deemed clinically relevant [34]. Therefore, 100 patients in each arm (allowing for a 20% dropout over 52 weeks) will be sufficient to detect the differences between the treatment and placebo groups. The participants will be recruited from 9 study sites, and competitive recruitment strategy will be implemented.

**Table 2:** Sample size calculation

	Mean (SD)	Detectable difference	Calculated sample size (per arm)
Suprapatellar pouch effusion (cm^2^)	2.0 ± 1.5	35%	74
VAS knee pain (0–100)	50 ± 20	20%	64

### Statistical analysis plan

Intention-to-treat and per-protocol (for patients who have taken > 80% the study medication) analyses of primary and secondary outcomes will be performed. The changes in outcome measures among all randomised participants at 52 weeks will be used for intention-to-treat analyses. Independent *t* tests and chi-square tests will be used to examine baseline characteristics of participants between groups. General linear mixed models to account for the effects of clustering by trial sites will be used to compare changes in outcomes in univariable and multivariable modelling between groups. Additional adjustment for imbalanced baseline factors will be made for further analyses. Baseline data will be used for data imputation assuming missing at random. A 95% confidence interval not including the null point or a *p*-value < 0.05 (two-tailed) will be considered statistically significant.

### Withdrawal

All participants are able to withdraw at any time of the trial. The time and reason will be recorded. If participants withdraw after 6 months of follow-up, we will attempt to obtain their final MRI scans and pain levels.

### Oversight and monitoring

The chief investigator, project manager, enrolling research staff, and three independent Clinical Research Associates from the Clinical Research Centre of Zhujiang Hospital comprise the Operation Committee (OC) at the coordinating centre. The OC will have weekly meeting and audit trial conduction in each institution for at least three times a year. Patient recruitment and data quality will also be checked regularly. The PIs, the consultants, and the biostatistician comprise the Trial Steering Committee (TSC), which provides supervision of trial development and oversees trial progress. The frequency of TSC meetings will be determined by PIs and the funder. There is no specific stakeholder and public involvement group in the current study. The Data Monitoring Committee (DMC) consists of a statistician, two research doctors, the CI, and an independent chair without any competing interests. The DMC will oversee data integrity, security, and efficacy; guide blinded primary data analyses; and report study results.

### Data integrity and management

All data obtained will be kept strictly confidential and will be stored electronically on a database with secured and restricted access. Participants will be numbered randomly, and other information capable of identifying individuals will be removed. There will be no planned interim analyses and stopping guidelines. The DMC will have access to the original study database.

## Discussion

This protocol is aimed to determine if MTX intervention can relieve pain symptom and slow disease progression in symptomatic knee OA patients with an inflammatory phenotype. Similar to its effect on RA, MTX may have beneficial effects for synovitis and effusion in OA patients, which would be an important strategy for the treatment of mid- to late-stage OA if being proven. Our protocol aims to perform a well-designed multicentre randomised controlled trial with a longer follow-up period to assess structural changes using sensitive and objective measurements over the course of knee OA. We will also be able to determine how acceptable MTX is used in those with knee OA.

Synovitis can affect OA progression through pro-inflammatory mechanisms. Preliminary studies have reported that synoviums are an important source of cytokines within joints. Local synovial levels of pro-inflammatory cytokines such as IL-1β, TNF-α, and IL-6 are detectable even in early OA [35] and have been implicated in disease progression and joint pain of OA [36–38]. Some in vitro and animal studies have documented that these cytokines can enhance cartilage degradation or induce bone resorption [39–41]. A cohort study reported that higher baseline IL-6 levels were associated with greater radiographic OA, and predicted greater loss of knee cartilage volume [42]. Baseline circulating TNF-α and change in TNF-α over 2.5 years were associated with increased knee pain over 5 years in older adults [37]. Circulating levels of C-reactive protein (CRP) are elevated in OA and are associated with increased disease progression [43, 44]. Baseline circulating CRP and change in CRP over 2.5 years were associated with increased knee pain over 5 years [37]. Our meta-analysis showed that serum CRP levels in OA were modestly but statistically significantly higher than normal controls and were significantly associated with pain and decreased physical function [36].

In vitro, MTX inhibited the production of cytokines induced by T cell activation, including IL-6, IL-13, interferon (IFN)-γ, and TNF-α [45]. Oral intake of 10 mg MTX by RA patients led to marked inhibition of cytokine production in blood drawn after 2 h [45]. MTX treatment (up to 15 mg for 24 weeks [46] or mean 8.7 mg for 1 year [15]) resulted in significant reductions of circulating IL-6, sIL-2R, and/or CRP. MTX (15 mg/week for 1 year) for the treatment of early RA significantly decreased disease activity as well as serum CRP and ESR levels [47]. In patients with RA, treatment with MTX reduced area of knee synovitis at the suprapatellar pouch by 35% [33]; inhibited inflammatory response in synovial tissue, including reducing numbers of macrophage, and suppressing expressions of intercellular adhesion molecule 1, IL-1, TNF-α, and CRP [48]; and decreased BMLs and synovitis even at very early stage [49].

Therefore, we propose MTX for the treatment of mid- to late-stage knee OA with the aim to reduce local and systemic inflammatory cytokines, to control patients’ joint inflammation, to alleviate knee pain, and to delay the time of joint replacement. In view of the side effect of MTX and the study population which are mainly older adults, we will give folic acid tablets to reduce the side effects and pay close attention to the patients who cannot tolerate MTX. In previous studies, MTX was used at dosages of 20–25 mg/week for 6 months to test if it was effective to reduce symptoms and disease progression in knee OA patients [19]. In our study, we will choose the highest dosage of 15 mg/week and the total course of 52 weeks, with consideration of the ethnic differences and slow-acting property of this drug. MTX is a slow-acting drug, and the onset time is generally 3 to 6 months. We will assess the pain 6 times during the trial and measure MRI changes at baseline and 52 weeks. One year will be enough to observe the treatment effect on effusion-synovitis and Hoffa’s synovitis. In the future, joint replacement surgery and cartilage loss as the final OA outcomes could be recorded.

If the primary hypothesis of this RCT is proven, MTX has the potential to be used for slowing disease progression and reducing knee pain for inflammatory OA. It would significantly delay the time to knee joint replacement which would be of major public health importance. This suggests great potential for substantial cost savings through reductions in joint replacement surgery as well as the potential for great improvements in quality of life for those with OA. The proposed study will also determine the safety and acceptability of MTX with the potential of being a cost-effective and innovative approach to the management of knee OA.

## Data Availability

The datasets analysed during the current study will be available from the corresponding author on reasonable request.

## Abbreviations

OA: Osteoarthritis
KOA: Knee osteoarthritis
MTX: Methotrexate
MRI: Magnetic resonance imaging
WOMAC: Western Ontario and McMaster Universities Osteoarthritis Index
IPFP: Infrapatellar fat pad
OAI: Osteoarthritis Initiative
BML: Bone marrow lesion
VAS: Visual analogue scale
PHQ-9: Patient Health Questionnaire-9
SF-12: Short Form 12
ICC: Intraclass correlation coefficient
WORMS: Whole-Organ Magnetic Resonance Imaging Score
